# Unravelling Checkpoint Inhibitor Associated Autoimmune Diabetes: From Bench to Bedside

**DOI:** 10.3389/fendo.2021.764138

**Published:** 2021-11-05

**Authors:** Linda Wu, Venessa H. M. Tsang, Sarah C. Sasson, Alexander M. Menzies, Matteo S. Carlino, David A. Brown, Roderick Clifton-Bligh, Jenny E. Gunton

**Affiliations:** ^1^Centre for Diabetes, Obesity and Endocrinology, The Westmead Institute for Medical Research, Sydney, NSW, Australia; ^2^Department of Endocrinology, Royal North Shore Hospital, Sydney, NSW, Australia; ^3^Department of Endocrinology, Westmead Hospital, Sydney, NSW, Australia; ^4^Faculty of Medicine and Health, The University of Sydney, Sydney, NSW, Australia; ^5^Department of Immunology, Westmead Hospital, Sydney, NSW, Australia; ^6^NSW Health Pathology, Institute of Clinical Pathology and Medical Research (ICPMR), Sydney, NSW, Australia; ^7^Department of Medical Oncology, Royal North Shore Hospital, Sydney, NSW, Australia; ^8^Melanoma Institute Australia, The University of Sydney, Sydney, NSW, Australia; ^9^Department of Medical Oncology, Westmead Hospital, Sydney, NSW, Australia

**Keywords:** immune checkpoint inhibitor (ICI), diabetes mellitus, type 1 diabetes, immune related adverse events, immunotherapy

## Abstract

Immune checkpoint inhibitors have transformed the landscape of oncological therapy, but at the price of a new array of immune related adverse events. Among these is β-cell failure, leading to checkpoint inhibitor-related autoimmune diabetes (CIADM) which entails substantial long-term morbidity. As our understanding of this novel disease grows, parallels and differences between CIADM and classic type 1 diabetes (T1D) may provide insights into the development of diabetes and identify novel potential therapeutic strategies. In this review, we outline the knowledge across the disciplines of endocrinology, oncology and immunology regarding the pathogenesis of CIADM and identify possible management strategies.

## Introduction

The demonstrated successes of immune checkpoint inhibitors (ICIs) have resulted in a paradigm shift in the management of many malignancies. However, their association with novel immune related adverse effects (irAE), necessitates that a more detailed understanding of their pathogenesis is a major research priority. This will facilitate the early recognition and management of the autoimmune toxicities of ICIs that will become essential for many clinicians.

ICI-associated autoimmune diabetes mellitus (CIADM, also termed CPI-DM) is a novel form of autoimmune diabetes that arises as a rare complication of therapy, with an incidence between 0.2-1.4% (1–6). In contrast to many irAEs, CIADM often presents fulminantly with inexorable rapid progression (2–5). As with type 1 diabetes (T1D), the management is complex. We review the body of evidence across human and animal studies that add to our understanding of CIADM pathogenesis and islet autoimmunity in general. Finally, we highlight the clinical challenges in the management of patients with CIADM.

## Immune Related Adverse Events – an Overview

ICIs augment adaptive immunity *via* blockade of immune checkpoints that can be upregulated on exhausted/anergised T cells and/or manipulated by cancer cells to facilitate immune evasion. In doing so, ICIs can induce a potent anti-tumor immune response. The key agents in current use are monoclonal antibodies targeting cytotoxic T-lymphocyte associated protein 4 (CTLA-4), Programmed cell death protein 1 (PD-1) or its ligand Programmed cell death protein ligand 1 (PD-L1). The dramatic efficacy of ICIs was first demonstrated in metastatic melanoma in 2011 with the FDA approval of ipilimumab, an anti-CTLA4 monoclonal antibody (7). A 1 year overall survival of 25-35% with previous standard of care chemotherapy (8), increased to 73% with use of combination ICI therapies nivolumab (anti-PD1) and ipilimumab (anti-CTLA4) (9). ICIs provide a robust long term benefit with a 52% 5 year overall survival in patients with advanced melanoma after combination ipilimumab and nivolumab treatment (9). ICIs are now used as first or second line treatment in 17 solid tumors with 57 FDA approved indications, with eligibility expanding in USA from 1.54% of malignancies in 2011 to 43.63% in 2018 (10). In addition to metastatic malignancies, adjuvant ICI therapy with anti-PD1 has also been demonstrated to reduce risk of relapse in resected stage III or IV melanoma and resected renal cell carcinoma (11, 12).

One of the consequences of ICIs is the risk of developing irAEs. These can target virtually any organ system within the body and range in severity from mild to life-threatening. The incidence of grade 3-4 irAE (severe to life-threatening) is approximately 10-20% with anti-PD-1 monotherapy, 10-27% with anti-CTLA4 monotherapy, and 55% with combined anti-PD-1/CTLA-4 (9, 12–19). Time to irAE onset varies depending on the ICI type and the organ involved (20, 21). ﻿There does not appear to be a clear association between irAE and the underlying malignancy, with the exception of vitiligo which has a preponderance in melanoma patients, thought to relate to heightened reactivity between melanoma cells and melanocytic antigen targets in normal skin (22). Most (9, 23–25) but not all (26) studies have demonstrated that development of irAEs are associated with better treatment response, suggestive of a link between autoimmunity and anti-tumor immune responses. Interestingly, this association appears stronger in anti-PD-1 and anti-PD-L1 treated patients (27).

The mechanisms of irAE development remain ill-defined but appear specific to the target organ and ICI sub-type. Patterns of irAE seen with each class of ICI have revealed that specific immune checkpoints bear a more critical role in maintaining immune tolerance in certain organs. For example, ICI-related colitis had a 36% incidence in anti-CTLA4 treated patients in comparison to 1% in anti-PD1-treated patients (28). Furthermore, the key immune mediators appear to vary across irAE and vary compared to the matching classic *de novo* autoimmune diseases. One example is ICI related colitis, which is amongst the best studied of the irAE due to accessibility of tissue for histopathology. Colonic biopsies from patients with ICI related colitis demonstrated high levels of activated CD8+ T cells and relatively lower proportions of Treg cells in comparison to ulcerative colitis affected patients, indicating distinct immunological differences between the two diseases and also highlighting a key pathogenic role for T cells in this disease (29, 30). Contrastingly, histopathology from patients with ICI induced hypophysitis demonstrated both T and B cell infiltration with CTLA4 expression within pituitary cells and positive anti-pituitary antibodies in the circulation (31). Autoantibodies strongly associated with spontaneous autoimmune diseases such as T1D or myasthenia gravis are less commonly found in the irAE forms of disease (3, 32). Antibodies in CIADM will be discussed in detail below.

Amongst irAE, the most common endocrinopathies are thyroid dysfunction, hypophysitis and less commonly CIADM and adrenalitis. Whilst irAE such as ICI related colitis have been demonstrated to respond to immunosuppression (26), endocrinopathies do not appear to respond and result in irreversible hormonal deficiencies in the vast majority (24, 33, 34).

## ICI-Related Hyperglycemia

With increased reports of ICI related hyperglycemia and diabetes it is clear that a range of pathologies can contribute to elevated glucose. ICI-related autoimmune diabetes (CIADM) is the best described amongst these, largely due to its fulminant nature and thus high clinical importance. Other causes for ICI-related hyperglycemia include exacerbation of type 2 diabetes, steroid-induced hyperglycemia, pancreatitis with endocrine insufficiency, and autoimmune lipodystrophy (35–38). In one study of 411 patients receiving ICI therapy 27% had hyperglycemia, 33.3% of whom had pre-existing hyperglycemia, 39.5% had new-onset hyperglycemia associated with steroid use, none had CIADM and the remainder had an unclear precipitant (37).

Common causes of hyperglycemia should be excluded before making a diagnosis of CIADM, as the treatment varies widely. Similarly, readers should interpret with caution reported cases of CIADM patients to ensure the diagnosis was applied with definitive evidence of insulin deficiency or autoimmunity, rather than generic ICI-related hyperglycemia which may occur in the context of other therapies such as corticosteroids.

**Figure 1:**
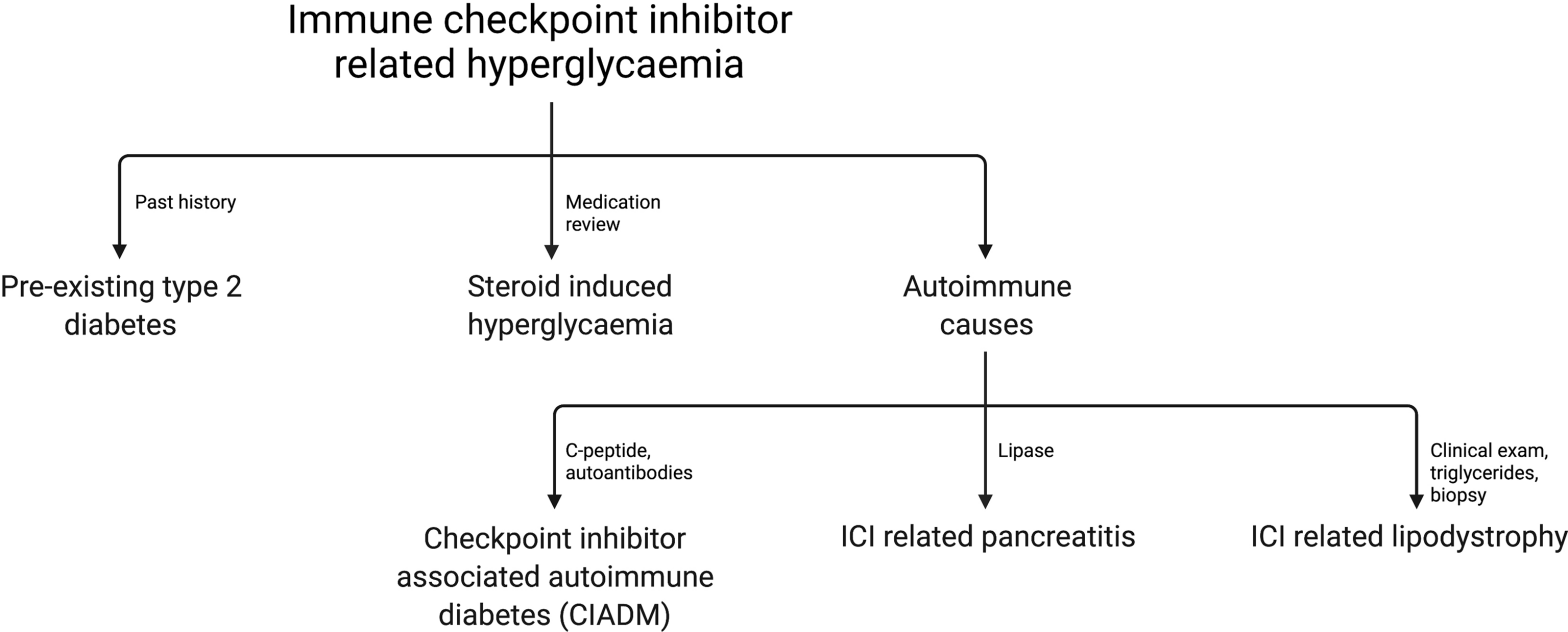
Flowchart of common differentials for ICI related hyperglycemia.

**Figure 1** outlines the most common causes of ICI related hyperglycemia and the key investigations to differentiate these.

## Pathophysiology of Checkpoint Inhibitor Associated Autoimmune Diabetes

### CIADM as a Novel Subtype of Type 1 Diabetes

Despite it being 100 years since the discovery of insulin and recognition of T1D, relatively little is known regarding the pathogenesis of islet autoimmunity. The commonly accepted theory is from Eisenbarth et al. in 1986, proposing that in a genetically predisposed individual, exposure to environmental triggers can precipitate islet autoimmunity and progressive β-cell destruction (39). Diagnosis of T1D requires demonstration of hyperglycaemia and is supported by evidence of autoimmunity and insulin deficiency, with 90% of patients being positive for islet autoantibodies at some point in their clinical course (40, 41). The common antibodies in T1D are to GAD (glutamic acid decarboxylase), insulin, ZnT8 (zinc transporter 8) and IA-2 (insulinoma-associated-2).

Over time distinct clinical phenotypes have been recognized within T1D. While traditional T1D is most commonly diagnosed in children and young adults, it can be diagnosed at any age. In addition, there is a milder, more slowly progressive phenotype in older patients termed latent autoimmune diabetes of adulthood (LADA) (41). ‘Fulminant T1D’ is increasingly reported in Asian populations, usually presenting with diabetic ketoacidosis. It is often associated with a lack of islet autoantibodies (42).

CIADM is considered a novel form of T1D, triggered specifically by ICI use. There is increasing evidence that the risk factors, pathophysiology and clinical phenotype of CIADM differ from traditional T1D.

### Defining Key Immune Mediators

There is robust evidence for the role of T cells as a key culprit in the development of T1D. T1D can be transferred by bone-marrow transplantation in humans, rats and mice. Pancreatic histopathology from patients with T1D typically demonstrates hallmark insulitis with immune infiltrates of predominately CD8+ T cells, and to a lesser extent macrophages, B cells and CD4+ T cells (43, 44). Islet immune infiltrates are typically limited in number and decline in frequency after diagnosis. Older patients and those with LADA display less insulitis than their classic younger T1D counterparts (45).

In contrast to T cells, the role of B cells in T1D is less well defined. Islet autoantibodies can predate onset of overt T1D by years (40). In genetically susceptible individuals the number of detectable islet autoantibodies directly correlates with risk of development of T1D (46). Whilst this evidence suggests islet antibody-producing B cells have some role in the pathogenesis of autoimmunity, islet autoantibodies do not display direct cytotoxicity to β-cells *in vitro* and they are not absolutely required for T1D development. That is demonstrated by a case of T1D in a person with X-linked agammaglobulinemia, lack of vertical transmission in autoantibody positive mothers with T1D and evidence that patients with hereditary B cell deficiencies may still develop T1D (47, 48).

The evidence delineating key immune mediators in CIADM is limited. To date only one patient with CIADM has had pancreatic histopathology reported, a 63 year old man with renal cell carcinoma and pre-existing type 2 diabetes whom developed diabetic ketoacidosis and low C-peptide after treatment with combination anti-CTLA4/PD-1 therapy (49). He was islet-antibody negative and his pancreas was resected due to tumor involvement. Histopathology demonstrated T cell infiltration throughout both endocrine and exocrine pancreas, with CD8^+^ T cell predominant insulitis. This finding suggests that CIADM may result as an off-target effect of ICI, given CD8^+^ T cells are the major cellular target of these drugs. Interestingly, few β-cells remained and PD-L1 was not expressed in those residual cells, despite their PD-L1 expression in classic T1D human pancreas (50). Although it is not possible to draw definitive conclusions, it is plausible that any PD-L1-positive β-cells were previously targeted for autoimmune destruction and thus absent by time of surgery.

The argument for T cell mediated β-cell destruction in CIADM is further supported by a small case series utilizing flow cytometry and tetramer assays on peripheral blood mononuclear cells from recently diagnosed CIADM patients (51). Hughes et al. identified an increased population of islet antigen specific CD8^+^ T cells in four CIADM patients, consistent with expected findings in new onset T1D patients (52, 53). The majority of these cells were CD45RO^+^ effector memory cells (51). Further studies are required to more clearly delineate the differences in immune changes between the fulminant process likely to be active in CIADM and T1D, where the autoimmune attack is thought to have preceded diagnosis by years (39).

Compared to traditional T1D where islet autoantibodies are present in 90% (40), autoantibody positivity is lower in CIADM, ranging from 0-71% (2–5, 54). The largest review thus far of CIADM patients reported anti-GAD positivity in 43% of the 151 cases tested (55). The relative paucity of traditional autoantibodies supports the theory that ICI triggered diabetes involves divergent immune pathways to those in traditional T1D islet antigen and B cell interactions. Six cases with CIADM have had retrospective testing of autoantibodies on pre-ICI treatment samples (2, 56–58). Of these patients, 3 patients (50%) had traditional T1D autoantibodies present on pre-treatment samples, 2 patients seroconverted to autoantibody positivity, and 1 patient remained negative for islet autoantibodies throughout (2, 56–58). This is a much higher incidence of autoantibody positivity than the general population, with the most common T1D autoantibody anti-GAD being present in 1.7% of the general population (59). These findings suggest that in a proportion of patients with CIADM, islet autoimmunity may predate ICI therapy. It may be tolerised *via* normal immune checkpoints but be unmasked by use of ICIs. Thus, traditional T1D autoantibodies do harbor some potential as biomarkers for CIADM, albeit limited in sensitivity by their low prevalence.

### PD-1/PD-L1 Axis

Exposure to ICI therapy involving the PD-1/PD-L1 axis is by far the strongest predictor for development of CIADM. Pharmacovigilance data from the FDA Adverse Events Reports System (FAERS) suggests that the incidence (as a proportion of all adverse events reported) is highest in those exposed to combination anti-CTLA4 plus either anti-PD-1 or anti-PD-L1 therapy (2.60%), followed by anti-PD-1 therapy alone (1.18%), anti-PD-L1 therapy alone (0.73%) and anti-CTLA4 therapy (0.33%) (60). The rare reports of CIADM with anti-CTLA4 monotherapy do not present clear evidence of either insulin deficiency or islet autoantibodies (60–62). This makes the diagnosis of CIADM less certain and the non-autoimmune differentials for hyperglycaemia still plausible in these cases.

The PD-1/PD-L1 axis has a well-established role in immune tolerance and maintenance of T cell anergy (63). PD-1 is an inhibitory molecule within the CD28 and CTLA4 superfamily and can be expressed on T cells, B cells, activated monocytes and dendritic cells. It interacts with two ligands, PD-L1 which is distributed across leukocytes, lymphoid and other tissues including pancreatic islets, and PD-L2 which is found on dendritic cells and monocytes. Polymorphisms in PD-1/PD-L1 genes in humans have been associated with a range of autoimmune diseases including type 1 diabetes, systemic lupus erythematosus and multiple sclerosis (64).

The development of CIADM after PD-1/PD-L1 inhibition highlights the critical role of this axis in the maintenance of self-tolerance towards pancreatic islets. The role of PD-L1 as a ‘defensive’ immunomodulator is supported by studies showing that β-cells from patients with T1D or autoantibody positive individuals express higher levels of PD-L1 compared to normal controls (50, 65), and this expression was further induced *in vitro* by type I and II interferons (65). Notably, PD-L1 expression was only found in islets containing β-cells and correlated with CD8^+^ T cell infiltration, implying PD-L1 expression in β-cells is upregulated in response to autoimmune attack (65). Similarly, peripheral blood findings have supported a role for PD-1 in T1D pathogenesis. Whole blood RNA analyses have demonstrated PD-L1 upregulation in a cohort of newly diagnosed patients with T1D (66). CD4^+^ expression of PD-1 was reduced in T1D patients, and CD4^+^ CD25^+^ T reg cells of patients with T1D have impaired upregulation of PD-1 in response to stimulation in comparison to normal controls, suggestive of a role for PD-1 in defective immune regulation even in traditional T1D pathogenesis (67–69). Higher frequency of CXCR5^-^ PD-1^hi^ CD4+ T peripheral helper cells is also present in T1D, a T-cell subtype implicated in chemotaxis and activation of B cells in autoimmune disease (70).

In the non-obese diabetic (NOD) model for autoimmune diabetes, mice null for either PD-1 or PD-L1 developed accelerated diabetes and significantly greater numbers of insulin specific T cells (71, 72). Interestingly, knockdown of PD-1/PD-L1 does not induce diabetes in other strains of mice such as C57BL/6, but instead induce lupus like disease and autoimmune cardiomyopathy, suggesting that PD-1/PD-L1 is not the only factor required to maintain islet tolerance and the relative importance of PD-1 may vary across both target organ and species (73, 74). Furthermore, PD-1/PD-L1 blockade broke islet tolerance and result in diabetes in NOD mice maintained on tolerising therapy with antigen specific splenocytes, whilst anti-CTLA4 and anti-PD-L2 did not (75). Similar to human studies, NOD mice showed increased PD-L1 expression in β-cells in the presence of IFN-gamma, insulitis and overt diabetes (50, 76). Conversely, loss of PD-1 in CD4^+^ T cells led to increased islet antigen specific immune infiltrate within islets, pancreatic lymph nodes and the spleen, as well as increased destructive insulitis (77).

### Genetic Risk

The role for genetic predisposition in T1D is well defined, with a 65% concordance in monozygotic twins diagnosed with T1D by age 60 (78). HLA polymorphisms are the strongest genetic risk factor, with class II haplotypes HLA-DR3-DQ2 and DR4-DQ8 seen in 90% of T1D patients (79, 80). In Asian populations DR4-DQ4 and DR9-DQ9 confer a high risk of T1D and fulminant diabetes (81). Genome-wide association studies have identified more than 50 further non-HLA susceptibility loci for T1D and these have contributed to the creation of genetic risk scores to aid in prediction of T1D and differentiation from other forms of diabetes (82).

Although the significance of HLA haplotypes has been pursued in CIADM, the findings have thus far been heterogenous. A recent review of 200 patients with CIADM showed that of the 78 patients with HLA genotyping reported, there was a pooled incidence of 51.3% that carried the HLA-DR4 haplotype, 14.1% had HLA-DR3 haplotype, whilst 10.3% had protective haplotypes (55). Meta-analysis demonstrated that presence of protective haplotypes was associated with a later median onset of CIADM (18 vs 9 weeks, p= 0.017). Only one case to date has had a T1D genetic risk score applied (58) and found a GRS score was below the 5^th^ percentile, indicating a lack of known genetic predisposition to T1D. Overall, HLA susceptibility haplotypes for classic T1D appear to have some bearing in CIADM, albeit a much weaker association than that seen in classic T1D, indicating other risk factors are in play.

### Role of the Exocrine Pancreas

Whilst T1D has traditionally been considered to involve isolated β-cell loss, there is increasing evidence that changes also occur in other islet cells and exocrine pancreatic tissue. Although β-cells only constitute 1-2% of pancreatic volume, even in recently diagnosed patients with T1D pancreatic volumes are smaller by approximately a third (83–85). Presence of these changes even in pre-symptomatic autoantibody positive patients suggests that the exocrine changes may play a role in disease pathogenesis rather than being purely secondary to hyperglycaemia. In fulminant T1D, elevations in lipase and amylase have also been reported at presentation (42). Conversely, in the classic T1D phenotype serum lipase and trypsinogen have also been shown to be significantly lower in patients with both T1D patients and patients positive for multiple autoantibodies in comparison to controls (86). Histology of pancreata from T1D patients shows immune cell infiltrate and fibrosis within exocrine tissue (43, 87), C4d complement deposition within exocrine ducts (88) and reduced acinar cell numbers (87). Although these changes do not result in overt exocrine insufficiency, patients with T1D have been shown to have lower fecal elastase values, in particular in those with established disease (89). It has been theorized that the development of these exocrine changes may be due to loss of the insulinotropic effect on acinar tissue, but this remains unproven. Another possible mechanism for damage is *via* either direct or bystander autoimmune attack, although the evidence for the former is limited to small scale studies of exocrine antigen targeted autoantibodies (90, 91).

Exocrine pancreatic injury after ICI therapy is common, with meta-analysis reporting 2.7% incidence of asymptomatic pancreatic enzyme elevation, and 1.9% incidence of overt pancreatitis (92). The true incidence of asymptomatic pancreatic enzyme elevation is likely even higher, with reports from a center performing routine lipase and amylase finding 8.4% had a grade 3 or higher elevated amylase and 26.9% grade 3 or higher lipase level (CTCAE v4) (93). Unlike CIADM, meta-analysis suggests pancreatitis is more common with anti-CTLA4 therapy (3.98%) compared to anti-PD-1 therapy (0.94%) (92). Abu-Sbieh et al. reported that of 2279 patients treated with ICIs, 4% developed pancreatitis (defined in this study by lipase with or without clinical symptoms) and of these, 7% developed diabetes – although the precise phenotype of diabetes in these patients is not clearly delineated (94).

Exocrine involvement in ICI related diabetes varies across a spectrum from overt pancreatitis with exocrine and endocrine insufficiency, through to a T1D-like phenotype with no features of pancreatic inflammation. The primary differentiating factor is likely to be the immune target of the irAE, which we postulate will be acinar tissue in the pancreatitis related diabetes phenotype and β-cells in the T1D-like phenotype. Overlap between phenotypes appears to be substantial, with a recent meta-analysis reporting that 51% of patients diagnosed with CIADM had an elevation in either lipase and/or amylase at time of diagnosis, a value disproportionately higher than that seen with ICI use in general (55). Rapid pancreatic atrophy has been reported in all CIADM patients whom have had pancreatic volumetry analyses, with significant decline from baseline pre-treatment volumes on imaging, through to CIADM diagnosis and follow-up (54, 95). The increased prevalence of exocrine pancreatic inflammation in CIADM patients raises the possibility of immune triggering, where the exposure of pancreatic epitopes through pancreatic inflammation and destruction increases immune sensitization and risk of islet autoimmunity.

Whilst it is apparent there exists a degree of overlap where patients may manifest features of both pancreatic inflammation and T1D as defined by sensitive biochemical parameters such as lipase, the extent to which these patients manifest clinical features of chronic pancreatitis and exocrine insufficiency remains unclear. One case series to date reported fecal elastase values in CIADM, with 2 of 5 patients demonstrating values consistent with pancreatic exocrine insufficiency (95).

### Enteroendocrine Involvement

Whilst the majority of T1D research focuses on β-cells, alpha cell mass is reduced in patients with longstanding T1D (96). This has correlated with findings of reduced glucagon responses in those with established T1D (97, 98), although glucagon responses in early T1D have been mixed (99, 100). The clinical implications of this are significant, as loss of glucagon from alpha cells compromises physiological defenses against hypoglycemia and increases morbidity and mortality (101, 102).

The impact of CIADM on alpha cell function is not well defined. Several case series have measured random glucagon levels in new onset CIADM patients and found no abnormalities (2, 3, 54). To further explore glucagon responses Marchand *et al*. performed mixed meal tests on 4 patients with fulminant presentations of CIADM, with 2 showing more blunted glucagon responses in comparison to 15 C-peptide negative longstanding T1D controls (95).

Given that incretins such as GLP-1 have roles in stimulating insulin secretion and suppressing glucagon, it is possible that dysregulation of incretins can contribute to dysglycaemia in CIADM. Bastin et al. demonstrated that patients with fulminant diabetes after ICI therapy have reduced GLP-1 and GIP levels at baseline and post oral glucose tolerance test in comparison to those with non-fulminant and type 2 diabetes (103). Small study numbers limit the conclusions that can be drawn on mechanisms and implications of impaired enteroendocrine function in CIADM.

### Putting It Together

**Table 1** summarizes the clinical and pathophysiological differences between CIADM and T1D.

**Table 1:** Comparison of the disease phenotype of checkpoint inhibitor associated autoimmune diabetes (CIADM) to traditional type 1 diabetes (T1D).

	CIADM	T1D
**Presentation**	DKA in 67.5% at presentation (55). 47.5% have a comorbid irAE, most common being thyroid (24.5%) (55).	DKA in 39% children at presentation (104), 6% in adults (105)
**Clinical course**	Fulminant presentation, median 9 weeks after ICI treatment	Progressive development of islet autoantibodies → overt hyperglycaemia at presentation
	No spontaneous remission phase or “honeymooning”. Overt insulin deficiency and low C-peptide at presentation in most (<0.3ng/ml in 63.4%) (55)	‘Honeymooning’ in 68.9% of children with T1D with partial recovery of β-cell function (106)
		Progressive decline in C-peptide, 48% maintain stimulated C-peptide >0.2nmol/L at 5 years (107)
**Autoantibodies**	Anti-GAD autoantibodies + in 43% (overall islet autoantibody positivity 20-71%) (55).	Islet autoantibodies + in 90% (40)
**Genetic predisposition**	65.4% with T1D susceptibility haplotype, 10.3% with T1D protective haplotype (55)	T1D susceptible haplotypes in 90% (80)
**Exocrine pancreas involvement**	Pancreatic enzymes elevated in 51% (55), pancreatic atrophy on imaging (54, 95)	Lower lipase vs normal controls except in fulminant phenotype (86), reduced pancreatic volumes (83–85)
**Proposed pathophysiology**	Prior exposure to environmental trigger leading to islet specific autoimmunity, tolerised by PD-L1	Genetic predisposed individual exposed to an environmental trigger, leading to autoimmune β-cell destruction
	Exposure to anti-PD1 or anti-PD-L1 unmasks autoimmunity and triggers β-cell destruction	

DKA, diabetic ketoacidosis; irAE, immune related adverse event.

**Figure 2:**
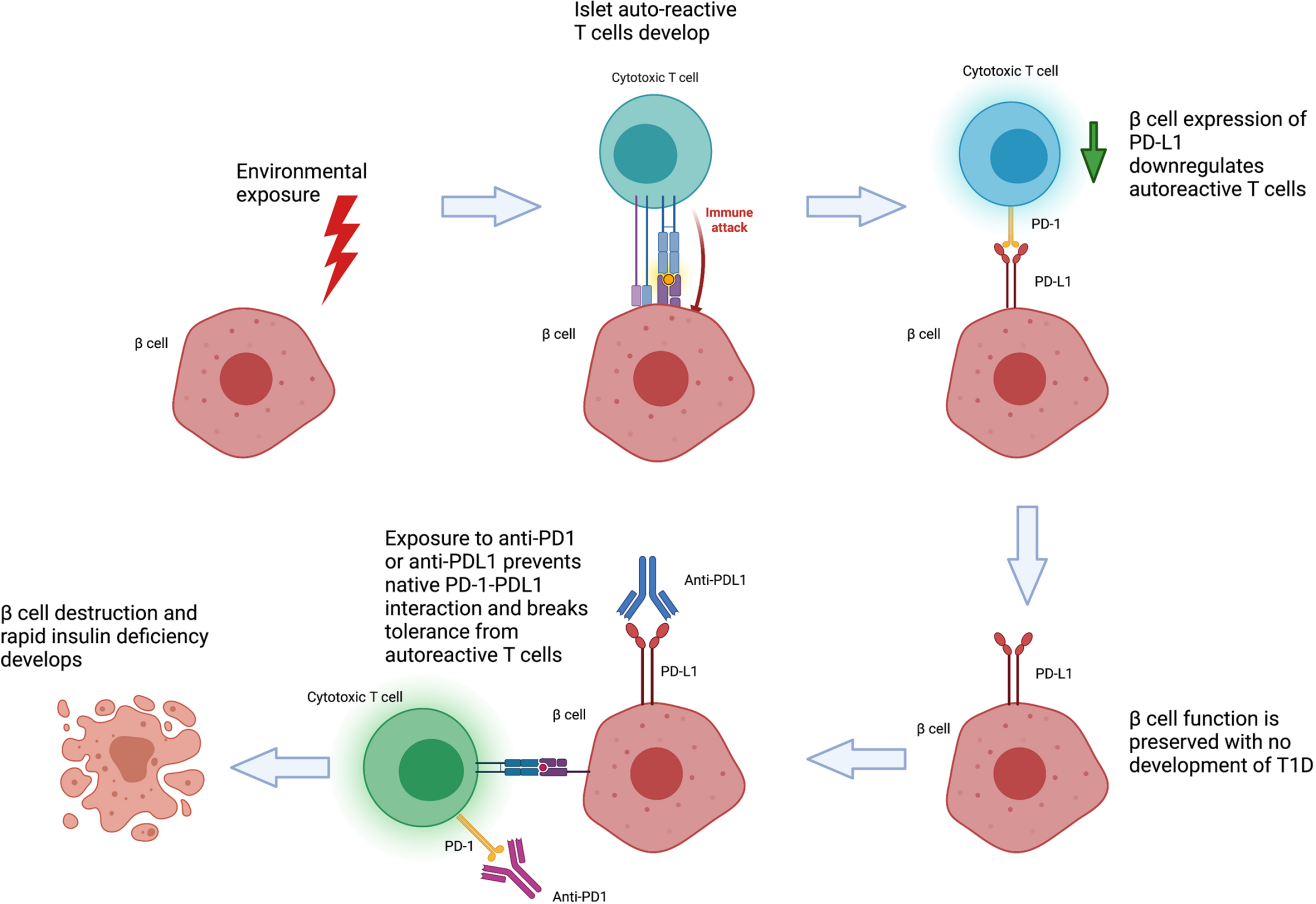
Proposed Pathogenesis of Checkpoint Inhibitor Associated Autoimmune Diabetes (CIADM).

On balance, the current literature supports a model of CIADM developing in genetically predisposed individuals who develop autoreactive T cells to beta-cells in response to an environmental trigger (**Figure 2**). These autoreactive T cells are generally controlled by immune checkpoints but result in pathology following their activation by anti-PD-1/PD-L1 therapy. Specific at-risk alleles for CIADM likely differ from classic T1D. Whilst patients with genetic susceptibility to impaired islet self-tolerance would have developed classic T1D earlier in life, populations with a particular reliance on the PD-1 axis for pancreatic tolerance may be at increased risk of CIADM specifically after anti-PD1/PD-L1 exposure. This may explain why such patients are able to remain free of T1D throughout adulthood until exposure to ICI therapy.

## Checkpoint Inhibitor Associated Autoimmune Diabetes

### Detection and Diagnosis

From case series to date, CIADM has an incidence of 0.2-1.4% in those treated with ICIs (1–6). In a FAERS database of 57,683 patients treated with ICIs with reported adverse events, a progressive increase in the proportion of cases of CIADM has been reported each year, likely reflective of the increased use of ICIs in general (60). As previously discussed, exposure to anti-PD-1 therapy is the greatest risk factor. No significant differences have been noted on meta-analysis when adjusting for age or sex (60), with the largest review reporting a median age of 64 years and male predominance (62.5%), reflective of the populations treated for melanoma and non-small cell lung cancer where ICIs are most commonly used (55). BMI is reported in a minority of cases. 50% (26 of 52) of subjects being of normal/low BMI, a value likely confounded by concurrent malignancy and toxicity. No strong link with a family history of diabetes has been noted, with 13% having a family history of either type 1 or 2 diabetes (55). Reflective of current ICI use, melanoma was the most common malignancy amongst subjects (50.5%), followed by lung cancer (26.0%) and renal cell carcinoma (7%) (55). In these patients, 47.5% were associated with another irAE, of which thyroid dysfunction was most common (24.5%) (55).

Diabetic ketoacidosis is a common presentation for CIADM, with incidence varying from 45.99-67.5% based on large cohort analyses (55, 60, 61). The hyperosmolar hyperglycemic state has been reported in 1% (55). The largest systematic review cohort to date of 200 patients reported a median time from ICI commencement to CIADM onset of 9 weeks and found this interval to be significantly shorter in patients presenting with DKA (8 weeks vs 15 weeks) (55). The most common symptoms at presentation are polyuria and polydipsia (48%), followed by gastrointestinal symptoms (vomiting, abdominal pain, diarrhea) in 41.7% and fatigue (40.6%) (55).

In terms of laboratory findings at presentation, hyperglycaemia was common with 94.3% having values **≥**300mg/dL (﻿**≥**16.7mmol/L) and a median HbA1c at presentation of 62mmol/mol (7.8%) (55). HbA1c was lower in those with shorter time from ICI commencement to CIADM onset, indicative of fulminant disease development in which the HbA1c is not a good indicator. In those with C-peptide testing within 1 month of diagnosis, C-peptide was overtly low in 63.4%. Islet autoantibodies were positive in 45%, with 43% being anti-GAD positive (55). HLA-DR4 or DR9 was identified in 65.3% (44 of 78) whilst 10.3% had traditionally protective alleles. Elevated pancreatic enzymes were present at diagnosis of CIADM in 51% and acute renal failure in 55% (55).

The interpretation of the above pooled data is limited by the heterogeneity in CIADM definition as determined by each case/series, and by reporting bias. To aid clinicians in the identification of the highest risk group of patients with ICI related diabetes and refine the future data that emerges for this disease we recommend the following diagnostic criteria for CIADM. Firstly presence of hyperglycaemia is required, either by random blood glucose **≥**11.1mmol or HbA1c **≥**6.5% [as per American Diabetes Association criteria for all forms of diabetes (41)], acknowledging that in fulminant presentations of CIADM HbA1c may not yet be elevated. Secondly, the suspicion of β-cell destruction needs to be demonstrated by presence a low C-peptide (<0.4nmol/L) soon after diagnosis. Seropositivity for 1 or more islet auto-antibodies (anti-GAD, anti-IA2, anti-insulin, anti-ZnT8) is supportive but not sufficient in isolation due to low overall prevalence in the CIADM population. Given the logistic challenges in performing a formal mixed meal test in CIADM patients we recommend a post-prandial C-peptide as an alternative, assessing for inappropriately low insulin production in a setting of relative hyperglycaemia. C-peptide adds value in the capture of those with rapidly progressive insulin deficient diabetes.

Trials in T1D demonstrate that presence of a detectable C-peptide is associated with improved outcomes and thus identifying patients with low C-peptide at presentation may capture a higher risk population that benefit from closer management (104). Case series suggest that C-peptide may not always be overtly low at diagnosis with CIADM (3, 54) and certainly in classic type 1 diabetes 48% of patients maintain a mixed meal stimulated C-peptide >0.2nmol/L in the first 5 years from diagnosis (105). Although even a normal C-peptide is considered inappropriate physiologically during hyperglycaemia, other forms of diabetes can also present with a normal C-peptide due to a pancreatic stunning effect with glucose toxicity (106) as well as reduced clearance of C-peptide in the setting of renal impairment. Thus, in those with a suspicion for CIADM diabetes but not yet manifesting an overtly low C-peptide at presentation we recommend repeating at 1 month to reduce the effect of these confounders.

**Figure 3:**
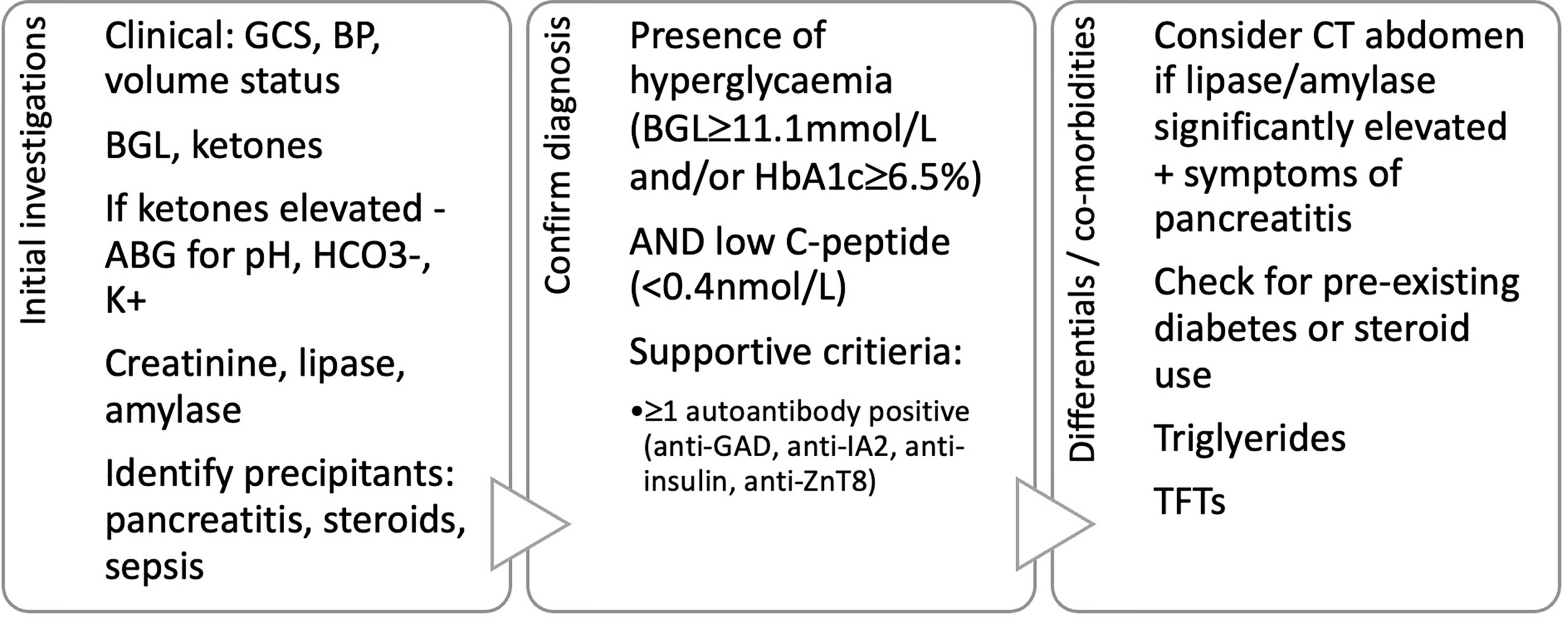
Proposed diagnostic criteria and initial investigations in patients presenting with hyperglycaemia after immune checkpoint inhibitor use. GCS, Glasgow coma scale; BP, blood pressure; BGL, blood glucose level; ABG, arterial blood gas; HCO3-, serum bicarbonate; K+, serum potassium; TFTs, thyroid function tests.

As depicted in **Figure 3**, in addition to the aforementioned tests for diagnosis of CIADM we recommend ancillary investigations to stratify the severity of the presentation, assess for need for intensive care support, identify precipitants and exclude differentials for ICI induced hyperglycaemia.

We acknowledge that these criterion will include patients with autoimmune pancreatitis related diabetes. There is significant overlap in the two populations and given the propensity of both towards diabetic ketoacidosis, they will require similar management with insulin. Those with concurrent significant pancreatitis require assessment for exocrine insufficiency with fecal elastase.

A further area of diagnostic challenge is the recognition of CIADM in patients with pre-existing type 2 diabetes. Various case reports have reported CIADM in patients with type 2 diabetes (5, 107). However, heterogenous criteria have been used to define CIADM in this setting, such as new insulin requirement, sudden worsening of HbA1c, positive islet auto-antibodies or loss of C-peptide. The challenge is in distinguishing the acuity and severity with which this occurs after ICI administration in comparison with the natural history of type 2 diabetes where eventual loss of C-peptide and insulin dependence may also occur. We have not sought to define diagnostic criteria for CIADM arising in patients with type 2 diabetes, as we feel this is an area where clinical judgement is paramount and ultimately both groups will benefit from insulin therapy.

A different ICI related cause of diabetes which should not be classified as CIADM is autoimmune lipodystrophy (35, 36). Acquired generalized lipodystrophy is characterized by autoimmune loss of adipose tissue, leading to severe insulin resistance, hypertriglyceridemia and non-alcoholic steatohepatitis. The two reported cases presented with severe hyperglycaemia and weight loss with notably elevated insulin, C-peptide and triglyceride levels. Both patients had been treated with anti-PD1 inhibitors. Diagnosis was confirmed with gluteal fat biopsy in both patients demonstrating panniculitis with extensive lymphoid infiltrate and fibrosis within adipose tissue (35, 36, 108–111). Triglycerides are included in routine ancillary investigations in ICI treated patients with hyperglycaemia and will assist in differentiating this condition, especially before clinically apparent changes in fat distribution are present.

### Clinical Course and Management

Management of CIADM requires insulin therapy, with all but two cases reporting a persistent and irreversible deficit in insulin production (2, 3, 54, 61). Two case reports have described spontaneous return to normal C-peptide levels and successful cessation of insulin therapy in patients with hyperglycaemia and positive islet autoantibodies. However, neither had documented low C-peptide at diagnosis so alternate diagnoses are possible (95, 112). There has been one case report of in a patient with newly diagnosed CIADM who required infliximab for treatment of concurrent oligoarthritis, with subsequent improvement in glycemic control and insulin cessation. This patient’s C-peptide levels were never overtly low and this case is confounded by steroid use (113). Use of corticosteroids with the intent to halt CIADM or other concurrent irAE is not effective (114–117). Overall, there is no current evidence to support use of immune suppression in CIADM.

As described by our group previously, patients with new-onset CIADM continuous glucose monitoring demonstrates similar patterns in glycemic variability to patients with T1D with no evidence of a ‘honey-moon’ period (3). This may reflect rapid β-cell loss as suggested by rapid decline in C-peptide levels. For this reason, we advocate that all patients should be managed akin to patients with T1D with use of basal bolus insulin or insulin pump therapy. Given the correlation between loss of C-peptide and hypoglycemia risk (118), we recommend early consideration of adjuncts like continuous glucose monitoring in those with low C-peptide to reduce hypoglycemia.

In contrast to traditional T1D, the oncological history and progress of a patient with CIADM bears significant implications both in prognosis and management goals. There is insufficient data available to draw conclusions on the impact of CIADM on oncological response. A recent review of 87 CIADM patients with reported oncological outcomes found a partial or complete response in 58.0%, which given 50.5% of the cohort had melanoma, is similar to the general ICI treatment cohort (55). Given the irreversibility of CIADM once it is diagnosed, cessation of ICIs for this reason is unlikely to be of benefit. Unlike T1D, CIADM affects a large spectrum of the adult population ranging from fit patients receiving adjuvant therapy to frailer patients already burdened by multiple lines of therapy and advanced disease. The risks of hypoglycemia are higher in frail populations, and more conservative glycemic targets are appropriate in those with poorer functional status and advanced progressive disease, with the individual in mind according to American Diabetes Association recommendations (41). In people who have short life-expectancy, insulin therapy should be simplified to target symptom control only. Conversely, it is also important to bear in mind that impressive survival outcomes offered by ICIs also signifies a larger population of patients will be cured and thus benefit from managing their diabetes with tighter glycemic targets to prevent long term glycemic complications. Clear communication regarding the expected cancer prognosis between oncologist and endocrinologist is key to setting safe management goals in this instance.

## Future Directions

Like all irAEs, the scope for further research into CIADM is broad. Further studies will help define the exact role of the exocrine pancreas and the extent to which acinar and other islet cells are affected. It is also unclear what predisposes a small subset of patients treated with anti-PD1/PD-L1 to this disease nor what other triggers may be required.

Biomarkers to predict whom amongst those treated with ICIs will develop CIADM would have high clinical utility in particular as indications for ICI use expand. Several biomarkers have shown utility in predicting irAE, ranging from autoantibodies (119–121), single nucleotide polymorphisms (122, 123), cytokines (124), lymphocyte count indices (125, 126) to microbiome analyses (127). Whilst autoantibodies can reliably predict T1D onset in traditional T1D, the relatively lower prevalence of autoantibodies suggests this is not the case in CIADM (2, 56–58). Given the unique immune trigger in CIADM, it is possible that novel autoantibodies to islet epitopes may exist that are yet undiscovered.

## Conclusion

As the use of ICIs continues to increase, the prevalence of CIADM will accordingly rise. Given the irreversible nature of the disease, further research to understand the pathophysiology and identify early biomarkers will be key to potentially preventing CIADM. Closer understanding of the presentation and initial investigations for CIADM amongst treating clinicians is essential to further reduce the incidence of fulminant DKA presentations and morbidity from this disease.

## Author Contributions

All authors listed have made a substantial, direct, and intellectual contribution to the work, and approved it for publication.

## Funding

AM is supported by Nicholas and Helen Moore and Melanoma Institute Australia. JG is supported by NHMRC Program Grant Complexity in Nutrition. The funders were not involved in the study design, collection, analysis, interpretation of data, the writing of this article or the decision to submit it for publication.

## Conflict of Interest

AM has served on advisory boards for BMS, MSD, Novartis, Roche, Pierre-Fabreand QBiotics. MC is an advisory board member for Amgen, BMS, Eisai, Ideaya, MSD, Nektar, Novartis, Oncosec, Pierre-Fabre, Qbiotics, Regeneron, Roche, Merck and Sanofi, and received honoraria from BMS, MSD, and Novartis.

The remaining authors declare that the research was conducted in the absence of any commercial or financial relationships that could be construed as a potential conflict of interest.

## Publisher’s Note

All claims expressed in this article are solely those of the authors and do not necessarily represent those of their affiliated organizations, or those of the publisher, the editors and the reviewers. Any product that may be evaluated in this article, or claim that may be made by its manufacturer, is not guaranteed or endorsed by the publisher.

## Abbreviations

ICI, immune checkpoint inhibitor; CIADM, checkpoint inhibitor associated autoimmune diabetes; T1D, type 1 diabetes; irAE, immune related adverse event.
